# The misery-is-not-miserly effect revisited: Replication despite opportunities for compensatory consumption

**DOI:** 10.1371/journal.pone.0199433

**Published:** 2018-06-27

**Authors:** Nitika Garg, Lisa A. Williams, Jennifer S. Lerner

**Affiliations:** 1 UNSW Business School, UNSW Australia, Sydney, New South Wales, Australia; 2 UNSW School of Psychology, UNSW Australia, Sydney, New South Wales, Australia; 3 Harvard University, Cambridge, Massachusetts, United States of America; Technion Israel Institute of Technology, ISRAEL

## Abstract

Sadness increases how much decision makers pay to acquire goods, even when decision makers are unaware of it. This effect is coined the “misery-is-not-miserly effect”. The paper that first established this effect is the second most-cited article appearing in *Psychological Science* in 2004. In light of its impact, the present study sought to assess whether the misery-is-not-miserly effect would replicate (a) in a novel context and (b) even when another way of alleviating a sense of loss (i.e., compensatory consumption) was available. Results revealed that the effect replicated in the novel context and, despite a prediction otherwise, even when individuals had an opportunity to engage in compensatory consumption. Moreover, a meta-analysis of the original effect and that observed in the present study yielded a small-to-medium effect (Cohen’s *d* = 0.43). As such, the present study lends evidentiary support to the misery-is-not-miserly effect and provides impetus for future research exploring the impact of sadness on consumer decision-making, specifically, and of emotion on decision processes, more generally.

## Introduction

Lerner, Small, and Loewenstein [1] discovered that sad individuals are willing to forego more money in exchange for a commodity than are individuals in a neutral state, a psychological phenomenon dubbed the “misery-is-not-miserly” effect [2]. Drawing on William James’s concept of “the material self,” Cryder et al. [2] predicted that self-oriented salience of loss might be the mechanism via which sadness impacts decision-making. Namely, the authors reasoned that sadness triggers an implicit goal to replace what has been lost, perhaps by purchasing new possessions. In line with this reasoning, Cryder et al. [2] found that self-focus mediated the relationship between sadness and buying price.

Despite several demonstrations of the misery-is-not-miserly effect [1, 2] and identification of self-focus as a mediator [2], many questions about the phenomenon remain. Perhaps the most central question about the misery-is-not-miserly effect is whether it is reliable, given that it runs counter to predictions from valence-based and mood-congruent theories of decision-making. According to these theories, negative-valenced states lead people to perceive objects in a globally pessimistic or devalued way (for reviews, see [3, 4, 5]). According to this line of reasoning, sadness–a negatively-valenced emotion–should depress valuations of prospective purchases, not increase them as observed empirically with the misery-is-not-miserly effect [1, 2]. Given that the misery-is-not-miserly effect runs counter to mood-congruent theories, it is important to test whether the effect replicates across contexts (e.g., with slight variations on emotion inductions and the choice price task). The present research aimed to do so.

Another key question about the misery-is-not-miserly effect stands in regard to its stability in the face of factors that might attenuate the impact of sadness on decision-making. If the effect of sadness on choice price arises as a form of compensatory consumption (for a review, see [6]) then perhaps the opportunity to consume something else (e.g., tasty food) might diminish the impact of sadness on choice price. At least one line of evidence lends support to this idea. Garg and Lerner [7] demonstrated that offering individuals the opportunity to engage in behaviors that increased their sense of control tempered sadness’ downstream impact on decision-making. Specifically, Garg and Lerner [7] found that offering participants an active choice alleviated the helplessness associated with sadness (see [8, 9]) and, in turn, attenuated the tendency for people in a sad state to over-consume comfort food. Other research also supports the control-enhancing (thus helplessness-reducing) impact of engaging in choice [10].

In the present research, we thus also sought to test whether compensating for the sense of loss (rather than helplessness) associated with sadness might temper the impact of sadness on decision-making. Prior research by Garg and colleagues has shown that sad individuals consume more hedonic food (e.g., buttered popcorn and candy) compared to those in neutral or happy affective states [11]. Tice, Bratslavsky, and Baumeister [12] also found negative affect increased consumption of unhealthful, fatty snack foods. These findings are consistent with a mood repair explanation [13]. We thus hypothesized that the opportunity to engage in compensatory consumption (e.g., eating buttered popcorn and drinking a beverage) would attenuate the misery-is-not-miserly effect.

## Materials and methods

### Overview

In the critical conditions of the original study [1], participants first watched either a sadness-inducing clip from *The Champ* or a control video from a National Geographic documentary. Participants then engaged in a choice price task.

The present study examined the effects of sadness on choice prices with a different set of emotion induction videos (clips from *Steel Magnolias* and *Blue Planet* to induce sadness relative to a neutral state, respectively) and a different commodity (a water bottle as opposed to a highlighter set). *Steel Magnolias* has been found to be effective in arousing sadness [14, 15, 16, 17, 18]. Similarly, water bottles have been used as commodities in prior consumer decision-making research stemming from the misery-is-not-miserly research [2]. In line with broader calls for replications in the field, establishing whether the misery-is-not-miserly effect replicates with these methodological changes represents an important test [19].

Key similarities and differences between the original study and the current research are presented in Table 1. Note that aside from the methodological changes described in Table 1 and the addition of a compensatory consumption opportunity in the consumption condition, all efforts were made to follow the paradigm of the original study. We report how we determined our sample size, as well as all data exclusions, manipulations, and measures in the study. Data and analyses scripts can be found on the Open Science Framework (https://osf.io/wsz2y/). This research was conducted with the approval of the Institutional Review Board at Carnegie Mellon University and meets the ethics criteria outlined by the American Psychological Association.

**Table 1:** Comparison between the present study and lerner et al. [1].

	Lerner et al. [1]	The present study
***Design and Methodology***		
Nature and setting	Lab experiment	Lab experiment
Sample size and type	199 university students (appx. 33/condition)	111 university students (appx. 27/condition)
Overall study design	3 (emotion) x 2 (task)	2 (emotion) x 2 (consumption opp.) x 1 (task)
	*Emotion*: sadness vs. disgust vs. neutral	*Emotion*: sadness vs. neutral
		*Consumption opportunity*: absent vs. present
	*Task*: choice price vs. sell price	*Task*: choice price
Emotion induction videos	*The Champ* (sadness)	*Steel Magnolias* (sadness)
	*Trainspotting* (disgust)	
	National Geographic documentary (neutral)	*Blue Planet* (neutral)
Commodity	Highlighter set	Water bottle
***Results–Sadness vs*. *Neutral***		
Choice price	*d* = 0.49	*d* = 0.40

### Participants

One hundred and eleven undergraduate student participants (60 female, 49 male, 2 gender not indicated; *M*_age_ = 21.47, *SD*_age_ = 3.70) completed the study in exchange for course credit. Sample size was determined via time constraints on data collection. After providing written informed consent, participants were seated in private cubicles equipped with computers and headsets, allowing no visual access to other participants.

### Design and procedure

Participants were randomly assigned to one of four conditions in a 2 (emotion: neutral vs. sad) x 2 (compensatory consumption opportunity: present vs. absent) design. Participants first completed the Positive and Negative Affect Schedule (PANAS; [20]) as a measure of baseline affect. Two things are of note: (1) PANAS items were interspersed with several other items not analyzed for the purposes of this replication, and (2) due to a programming error, the item “inspired” was not included. Responses on the 5-point scale were summed to form an index of baseline affect (α = .78). The former was used in an exploratory set of analyses controlling for baseline affect, mirroring Lerner et al. [1] and reported below. Participants next engaged in a video task, during which the manipulations occurred, and then completed measures of the dependent variables.

### Manipulations

According to assigned emotion manipulation condition, participants watched either a sadness-inducing clip from the film *Steel Magnolias* or a neutral clip from the nature documentary *Blue Planet*. Both videos were approximately 12 minutes in duration. While watching the videos, participants were either given the opportunity to engage in hedonic consumption or not according to compensatory consumption condition. Specifically, participants in the consumption opportunity conditions were offered buttered popcorn and a beverage under the guise of “better simulating a movie-going experience” (adapted from [11]). Note that in the prior research deploying this compensation opportunity, buttered popcorn was perceived as hedonic [11] (see Footnote i). The amount of popcorn each participant consumed was measured at the end of each session.

### Dependent variables

After the video task, participants next engaged in a choice price task. Barring the change in the commodity from a highlighter pen set to a water bottle, the choice protocol exactly replicated that used by Lerner and colleagues [1] with both, the current and the original studies using the Becker, DeGroot, and Marschak [21] price elicitation form. Specifically, participants were shown an insulated, re-usable water bottle and were then asked to indicate on a form whether they preferred the bottle or various cash amounts. The form consisted of 28 lines, each presenting the choice between the water bottle and an increasing cash amount ranging from $0.50 to $14.00, in $0.50 increments. For instance, Line 11 asked participants to indicate whether they preferred the water bottle or $5.50.

Responses on this task typically follow a pattern whereby participants prefer the water bottle over the listed cash amount up to a certain point, after which they flip to consistently indicating a preference for the cash amount. A ‘choice price’ in this case is the point at which the commodity is selected rather than cash; higher choice prices reflect a stronger desire to obtain the commodity. In order to incentivize true valuations and as per Lerner et al. [1], participants understood that, based on a pre-selected ‘price’ for the session, they would ultimately either receive cash or the water bottle at the conclusion of the experiment, according to their indicated choices. For instance, if the previously undisclosed ‘price’ for the water bottle was $5.50, participants would receive either the water bottle or the cash according to their selection on Line 11.

Finally, participants completed a suite of self-report questionnaires. One set of questions elicited their preference for activities such as watching a movie and talking to a friend (not analyzed here). They also reported whether they were currently dieting. This question was embedded within a set of questions regarding their eating behaviors before and during the experimental session. Next, participants indicated their current levels of sadness (“gloomy,” “sad,” “downhearted;” α = .94) on 9-point scales ranging from 0 (*did not experience the emotion at all*) to 8 (*experienced the emotion more strongly than ever before*), interspersed with several filler items assessing other affective states. Note that these items and the scale response were the same as used by Lerner et al. [1], except that “blue” in the original study was replaced with “gloomy” in the present study. One participant did not provide manipulation check data and was hence not included in the analysis of this variable. Participants then provided demographic information before being debriefed.

## Results

### Preliminary analyses

A 2 (emotion) x 2 (consumption opportunity) ANOVA on self-reported sadness revealed that the induction was successful with a significant main effect of emotion, *F*(1,106) = 211.34, *p* < .001, η^2^ = .67, 90% CI [.58, .72]. Participants who watched the sadness-inducing video (*M* = 4.43, *SD* = 1.80) reported higher levels of sadness than participants who watched the neutral video (*M* = 0.49, *SD* = 0.81; *d* = 2.82). As expected, this effect was not further qualified by consumption opportunity (*p* = .57), nor was the main effect of consumption opportunity significant (*p* = .08).

### Main analyses

Analysis of choice prices provided the critical test regarding the replication of the misery-is-not-miserly effect. Descriptive statistics per condition appear in Table 2. A 2 (emotion) x 2 (consumption opportunity) ANOVA revealed a main effect of emotion condition, *F*(1,107) = 4.34, *p* = .04, η^2^ = .04, 90%CI [.001, .11]. Consistent with Lerner et al. [1], participants who watched the sadness-inducing video (*M* = 6.42, *SD* = 2.74) indicated higher choice prices on average compared to participants who watched the neutral video (*M* = 5.35, *SD* = 2.72; *d* = 0.40). Contrary to the idea that the opportunity for compensatory consumption might diminish the robustness of the effect, having access to popcorn and a beverage did not impact choice prices (*p* = .89), nor did it qualify the effect of emotion (*p* = .37). Thus, we found that the misery-is-not-miserly effect replicated even in the face of an opportunity for compensatory consumption.

**Table 2:** Descriptive statistics for choice prices by condition.

	*Consumption Opportunity*	
	Absent	Present
***Emotion***		
Neutral	5.61 (2.52)	5.06 (2.96)
Sadness	6.22 (2.81)	6.62 (2.71)

Note. Standard deviation values appear in parentheses following mean values.

One reason why we did not observe a moderating effect of consumption opportunity might be due to the fact that not all participants, given the opportunity, actually consumed popcorn. Another reason for the null effect of the consumption condition could be that the dieters experienced the opportunity differently relative to non-dieters. In order to test these alternative explanations, two independent, exploratory ANOVAs excluding participants in the consumption conditions who reported that they were currently dieting (*n* = 6) or participants in the consumption conditions who did not consume any popcorn (*n* = 7), were run on choice prices. The exclusions based on these two criteria were non-overlapping. These analyses revealed findings similar to those reported above. In both, significant main effects of emotion condition (*p*s < .03) on choice prices emerged. These main effects were not further qualified by interactions between emotion and consumption condition (*p*s > .23), nor did a main effect of consumption condition emerge (*p*s > .79). Effect sizes for the emotion effects on choice prices (*d* = 0.44, 0.41, respectively) in these exploratory analyses were comparable to the main analyses.

As a further check, we ran the planned analyses (full sample) with baseline affect as a covariate, following Lerner et al. [1], Footnote 3. All reported results regarding the impact of emotion condition and consumption condition remained unchanged in terms of statistical significance. Thus, we conclude that the effect replicates and is consistent, even after accounting for participants who might not have engaged in compensatory consumption or might not have reacted to the consumption opportunity as people from the general population (non-dieters) would.

### Meta-analysis

In order to assess the overall robustness of the misery-is-not-miserly-effect, we carried out a meta-analysis of the effect size of sadness versus neutral on choice prices from Lerner et al. [1] and the present study. Following current recommendations [22], we meta-analyzed the two effects using a fixed effects approach, in which the mean effect size (in *r*) was weighted by sample size. Raw means were used to calculate relevant effect sizes. Overall, the meta-analyzed effect was significant and non-zero (*M*_r_ = .21, 95% CI [.06, .36], *Z* = 2.80, *p*_two-tailed_ = .005), such that sadness led to higher choice prices compared to a neutral state. Conversion to Cohen’s *d* resulted in a meta-analyzed effect size estimate of 0.43 with a 95% confidence interval ranging from 0.12 to 0.77. We note that a fully random effects test of the meta-analyzed effect produced a nearly identical estimate of the effect that was marginally significant (*M*_r_ = .22, *t*(1) = 8.60, *p*_two-tailed_ = .07). However, as noted by Goh et al. [22], the fully random effects approach is highly conservative with small numbers of studies, as is the case here.

## Discussion

The close conceptual replication and extension study reported here sought to examine the misery-is-not-miserly effect documented by Lerner et al. [1]. Overall, results replicated the effect: sadness increased choice prices. These results were obtained with variations in methodology, affording confidence in the underlying reliability of the effect [19]. Moreover, a meta-analysis of the original effect and that observed in the present study resulted in an effect size estimate with a confidence interval not including zero, further lending evidentiary support for the misery-is-not-miserly effect.

The current study also tested whether the opportunity for compensatory consumption would attenuate or negate the impact of sadness on choice prices. Results did not support this possibility: the misery-is-not-miserly effect emerged even among participants who had the opportunity to engage in hedonic consumption. This finding provides theoretical insight into the nature of sadness and its impact on decision-making. Prior literature has found consistent support for sadness’ appraisal theme of loss and helplessness [8, 9]. In line with such appraisals, sadness results in compensatory consumption (e.g., hedonic food consumption), in a bid to regulate the negative state [12, 14]. Prior research that has shown that addressing the sense of helplessness innate to sadness via engaging in choice such as in shopping [23] decouples the sadness-choice price link [7]. Leveraging such findings, we predicted that hedonic consumption, by addressing the sense of loss, would similarly moderate the misery-is-not-miserly effect. This prediction was not supported, raising the possibility that sadness’ impact on decision-making is driven by its characteristic sense of helplessness, and not loss. Future research might test this hypothesis via studies that both measure and manipulate the proposed mechanisms [24].

Of course, a single study enabled testing the effect under only selected parameters, further constrained by our aim to conduct a close conceptual replication. We acknowledge that given the limited inferences that can be drawn from a single study, the null effects obtained for the moderating effect of compensatory consumption should be put to further empirical test. Future research on the misery-is-not-miserly effect might also consider the impact of other operationalizations of sadness, hedonic consumption, and choice price. Further, it will certainly be important for future work to undertake highly-powered replications [25] that recruit more diverse samples than undergraduate students from North America [26]. One promising route for such efforts would be to adopt a multi-lab approach [27, 28].

Moreover, in line with a broader goal to establish both commonalities and differences among discrete emotions, it is pivotal that future research include comparisons to other negative emotions (e.g., anger, [23]; disgust, [1]) as well as positive emotions (e.g., pride and gratitude). Another fruitful avenue for future research will be to explore the conditions under which sadness, and indeed other emotions, impact other types of consumer decision-making (e.g., attitudes and brand preferences [29, 30]).

Overall, this work underscores the importance of investigating the role of discrete emotions, corroborating past research across a variety of sub-disciplines of psychology [3, 31, 32]. In conclusion, it appears that misery is indeed *not* miserly, and robustly so.

## References

[1] Lerner JS, Small DA, Loewenstein G. Heart strings and purse strings: Carryover effects of emotions on economic decisions. Psychol Sci. 2004;15(5): 337–341. doi: 10.1111/j.0956-7976.2004.00679.x 15102144 10.1111/j.0956-7976.2004.00679.x

[2] Cryder CE, Lerner JS, Gross JJ, Dahl RE. Misery is not miserly: Sad and self-focused individuals spend more. Psychol Sci. 2008;19(6): 525–530. doi: 10.1111/j.1467-9280.2008.02118.x 18578840 10.1111/j.1467-9280.2008.02118.x PMC4142804

[3] Keltner D, Ellsworth PC, Edwards K. Beyond simple pessimism: Effects of sadness and anger on social perception. J Pers Soc Psychol. 1993;64(5): 740–752. doi: 10.1037/0022-3514.64.5.740 8505705 10.1037//0022-3514.64.5.740

[4] Keltner D, Lerner JS. Emotion In: Gilbert DT, Fiske ST, Lindzey G, editors. The handbook of social psychology. New York, NY: Wiley; 2010 pp. 317–52. doi: 10.1002/9780470561119.socpsy001009

[5] Lerner JS, Li Y, Valdesolo P, Kassam KS. Emotion and decision making. Annu Rev Psychol. 2015;66: 799–823. doi: 10.1146/annurev-psych-010213-115043 25251484 10.1146/annurev-psych-010213-115043

[6] Rucker DD, Galinsky A. Compensatory consumption In Ruvio AA, Belk RW, editors. The Routledge Companion to Identity and Consumption. Routledge Taylor & Francis Group; 2012 pp. 207–215. doi: 10.4324/9780203105337.ch21

[7] Garg N, Lerner JS. Sadness and consumption. J Consum Psychol. 2013;23(1): 106–113. doi: 10.1016/j.jcps.2012.05.009

[8] Keltner D, Locke KD, Audrain PC. The influence of attributions on the relevance of negative feelings to personal satisfaction. Pers Soc Psychol Bull. 1993;19(1): 21–29. doi: 10.1177/0146167293191003

[9] Lazarus RS. Emotion and adaptation London, UK: Oxford University Press; 1991.

[10] Rick SI, Pereira B, Burson KA. The benefits of retail therapy: Making purchase decisions reduces residual sadness. J Consum Psychol. 2014;24(3): 373–380. doi: 10.1016/j.jcps.2013.12.004

[11] Garg N, Wansink B, Inman JJ. The influence of incidental affect on consumers' food intake. J Mark. 2007;71(1): 194–206. doi: 10.1509/jmkg.71.1.194

[12] Tice DM, Bratslavsky E, Baumeister RF. Emotional distress regulation takes precedence over impulse control: If you feel bad, do it!. J Pers Soc Psychol. 2001;80(1): 53–67. doi: 10.1037/0022-3514.80.1.53 11195891

[13] Andrade EB. Behavioral consequences of affect: Combining evaluative and regulatory mechanisms. J Consum Res. 2005;32(3): 355–362. doi: 10.1086/497546

[14] Britton JC, Taylor SF, Berridge KC, Mikels JA, Liberzon I. Differential subjective and psychophysiological responses to socially and nonsocially generated emotional stimuli. Emotion. 2006;6(1): 150–155. doi: 10.1037/1528-3542.6.1.150 16637758 10.1037/1528-3542.6.1.150

[15] Puccinelli NM, Deshpande R, Isen AM. Should I stay or should I go? Mood congruity, self-monitoring and retail context preference. J Bus Res. 2007;60(6): 640–648. doi: 10.1016/j.jbusres.2006.06.014

[16] Kreibig SD, Wilhelm FH, Roth WT, Gross JJ. Cardiovascular, electrodermal, and respiratory response patterns to fear‐and sadness‐inducing films. Psychophysiology. 2007;44(5): 787–806. doi: 10.1111/j.1469-8986.2007.00550.x 17598878 10.1111/j.1469-8986.2007.00550.x

[17] Gross JJ, Levenson RW. Hiding feelings: The acute effects of inhibiting negative and positive emotion. J Abnorm Psychol. 1997;106(1): 95–103. doi: 10.1037/0021-843X.106.1.95 9103721 10.1037//0021-843x.106.1.95

[18] Fredrickson B L., Levenson RW. Positive emotions speed recovery from the cardiovascular sequelae of negative emotions. Cogn Emot. 1998;12(2): 191–220. doi: 10.1080/026999398379718 21852890 10.1080/026999398379718 PMC3156608

[19] Earp BD, Trafimow D. Replication, falsification, and the crisis of confidence in social psychology. Front Psychol. 2015;6: 621 doi: 10.3389/fpsyg.2015.00621 26042061 10.3389/fpsyg.2015.00621 PMC4436798

[20] Watson D, Clark LA, Tellegen A. Development and validation of brief measures of positive and negative affect: The PANAS scales. J Pers Soc Psychol. 1988;54(6): 1063–1070. doi: 10.1037/0022-3514.54.6.1063 3397865 10.1037//0022-3514.54.6.1063

[21] Becker GM, DeGroot MH, Marschak J. Measuring utility by a single-response sequential method. Syst Res Behav Sci. 1964;9(3): 226–232. doi: 10.1002/bs.3830090304 10.1002/bs.3830090304 5888778

[22] Goh JX, Hall JA, Rosenthal R. Mini meta-analysis of your own studies: Some arguments on why and a primer on how. Soc Personal Psychol Compass. 2016;10(10): 535–549. doi: 10.1111/spc3.12267

[23] Rick SI, Pereira B, Burson KA. The benefits of retail therapy: Making purchase decisions reduces residual sadness. J Consum Psychol. 2014;24(3): 373–380. doi: 10.1016/j.jcps.2013.12.004

[24] Pirlott AG, MacKinnon DP. Design approaches to experimental mediation. J Exp Soc Psychol. 2016;66: 29–38. doi: 10.1016/j.jesp.2015.09.012 27570259 10.1016/j.jesp.2015.09.012 PMC4999253

[25] Anderson SF, Kelley K, Maxwell SE. Sample-size planning for more accurate statistical power: A method adjusting sample effect sizes for publication bias and uncertainty. Psychol Sci. 2017;28(11): 1547–1562. doi: 10.1177/0956797617723724 28902575 10.1177/0956797617723724

[26] Henrich J, Heine SJ, Norenzayan A. The weirdest people in the world? Behav Brain Sci. 2010;33(2–3): 61–83. doi: 10.1017/S0140525X0999152X 20550733 10.1017/S0140525X0999152X

[27] Klein RA, Ratliff KA, Vianello M, Adams RB, Bahník Š, Bernstein MJ, et al Investigating variation in replicability: A “many labs” replication project. Soc Psychol. 2014;45(3): 142–52. doi: 10.1027/1864-9335/a000178

[28] Ebersole CR, Atherton OE, Belanger AL, Skulborstad HM, Allen JM, Banks JB, et al Many Labs 3: Evaluating participant pool quality across the academic semester via replication. J Exp Soc Psychol. 2016;67: 68–82. doi: 10.1016/j.jesp.2015.10.012

[29] Sela A, Wheeler SC, Sarial-Abi G. We are not the same as you and I: Causal effects of minor language variations on consumers' attitudes toward brands. J Consum Res. 2012;39(3): 644–661. doi: 10.1086/664972

[30] Sengupta J, Fitzsimons GJ. The effects of analyzing reasons for brand preferences: disruption or reinforcement?. J Mark Res. 2000;37(3): 318–330. doi: 10.1509/jmkr.37.3.318.18776

[31] Shiota MN, Campos B, Oveis C, Hertenstein MJ, Simon-Thomas E, Keltner D. Beyond happiness: Building a science of discrete positive emotions. Am Psychol. 2017;72(7): 617–643. doi: 10.1037/a0040456 29016167 10.1037/a0040456

[32] Lench HC, Flores SA, Bench SW. Discrete emotions predict changes in cognition, judgment, experience, behavior, and physiology: A meta-analysis of experimental emotion elicitations. Psychol Bull. 2011;137(5): 834–855. doi: 10.1037/a0024244 21766999 10.1037/a0024244

